# Crystal structure and Hirshfeld surface analysis of 1-(di­methyl­amino­meth­yl)-2-(pyrrolidin-1-ylmeth­yl)ferrocene complexes with zinc(II) bromide and cadmium(II) bromide

**DOI:** 10.1107/S2056989026004688

**Published:** 2026-05-12

**Authors:** Beata Moritz, Tristan Mairath, Carsten Strohmann

**Affiliations:** a TU Dortmund University, Department of Chemistry and Chemical Biology, Inorganic Chemistry, Otto-Hahn-Strasse 6, 44227 Dortmund, Germany; University of Neuchâtel, Switzerland

**Keywords:** crystal structure, ferrocene derivatives, zinc bromide, cadmium bromide, transition metal complexes, Hirshfeld surface analysis

## Abstract

Two transition-metal complexes, *rac*-di­bromido­[1-(di­methyl­amino­meth­yl)-2-(pyrrolidin-1-ylmeth­yl)ferrocene]zinc(II), [FeZnBr_2_(C_5_H_5_)(C_13_H_22_N_2_)] (*rac*-**1**), and *rac*-di­bromido­[1-(di­methyl­amino­meth­yl)-2-(pyrrolidin-1-ylmeth­yl)ferrocene]cadmium(II), [FeCdBr_2_(C_5_H_5_)(C_13_H_22_N_2_)] (*rac*-**2**), crystallize in the form of yellow blocks and were characterized by single-crystal X-ray diffraction. They were synthesized using *rac*-1-(di­methyl­amino­meth­yl)-2-(pyrrolidin-1-ylmeth­yl)ferrocene (*rac*-**3**) as starting material, which is characterized here for the first time. Both, the zinc center in complex *rac*-**1** and the cadmium center in complex *rac*-**2** exhibit a tetra­hedral coordination geometry. Nevertheless, there are some differences in terms of the bond lengths as well as the bond angles. Furthermore, not only the space groups, *P*2_1_/*n* for *rac*-**1** and *P*2_1_2_1_2_1_ for *rac*-**2**, but also the crystal packings differ from each other, which can be seen in different configurations of the pyrrolidine substituents. To investigate the inter­molecular inter­actions leading to these structural differences, Hirshfeld surface analyses were performed. They showed that H⋯H inter­actions make the largest contribution to the crystal packing in both structures, with 69.0% and 66.6% for *rac*-**1** and *rac*-**2**, respectively.

## Abstract 2

The title transition-metal complexes *rac*-**1** and *rac*-**2** exhibit some differences in terms of the bond lengths as well as the bond angles. Furthermore, not only the space groups, *P*2_1_/*n* for *rac*-**1** and *P*2_1_2_1_2_1_ for *rac*-**2**, but also the crystal packings differ from each other, which can be seen in different configurations of the pyrrolidine substituents.

## 1. Chemical context

Functionalized ferrocenes are widely applied in catalytic transformations. Therefore, suitable methods for the synthesis of different ferrocene derivatives are important (Schaarschmidt & Lang, 2013 ▸). An example of a suitable starting material for such derivatization is *N*,*N*-di­methyl­amino­methyl­ferrocene. Starting from this compound, it is possible to synthesize 1,2-disubstituted ferrocenes by li­thia­tion and subsequent substitution of the *ortho*-position, which is preferred due to the D*o*M effect (*Directed ortho Metalation*) originating from the amino group (Marr, 1967 ▸). In the presence of substoichiometric amounts of the chiral auxiliary (*R*,*R*)-tetra­methyl-1,2-cyclo­hexa­nedi­amine (TMCDA), an enanti­oselective synthesis with high stereoselectivities up to >99:1 is possible, besides a racemic li­thia­tion (Steffen *et al.*, 2013 ▸). In this work, a 1,2-disubstituted ferrocene in its racemic form is reported. Compound *rac*-**3** was synthesized by *ortho*-li­thia­tion using *tert*-butyl­lithium and addition of 1-methyl­idenepyrrolidin-1-ium chloride as an electrophile. The synthesis and characterization of this di­amino ferrocene *rac*-**3** is reported here for the first time. It has been shown to be a suitable ligand for the formation of transition-metal complexes.

In addition to ferrocene ligands, other ligands are able to form transition-metal complexes that can be used as catalysts in a variety of synthetically relevant reactions. For example, it has been reported that certain cadmium(II) complexes with oxazoline-based ligands catalyze C—N cross-coupling reactions (Jia *et al.*, 2015 ▸). In addition, halogen-bonded zinc(II)– and cadmium(II)–aryl­hydrazone complexes exhibit catalytic activity in cyclo­addition reactions of CO_2_ with epoxides (Aliyeva *et al.*, 2023 ▸). Furthermore, di­amine zinc complexes can be used as catalysts in lactide polymerization (Eckert *et al.*, 2013 ▸). In this work, a zinc(II) complex, *rac*-**1**, and a cadmium(II) complex, *rac*-**2**, could be crystallized, after the reaction of ligand *rac*-**3** with the corresponding bromide salts.
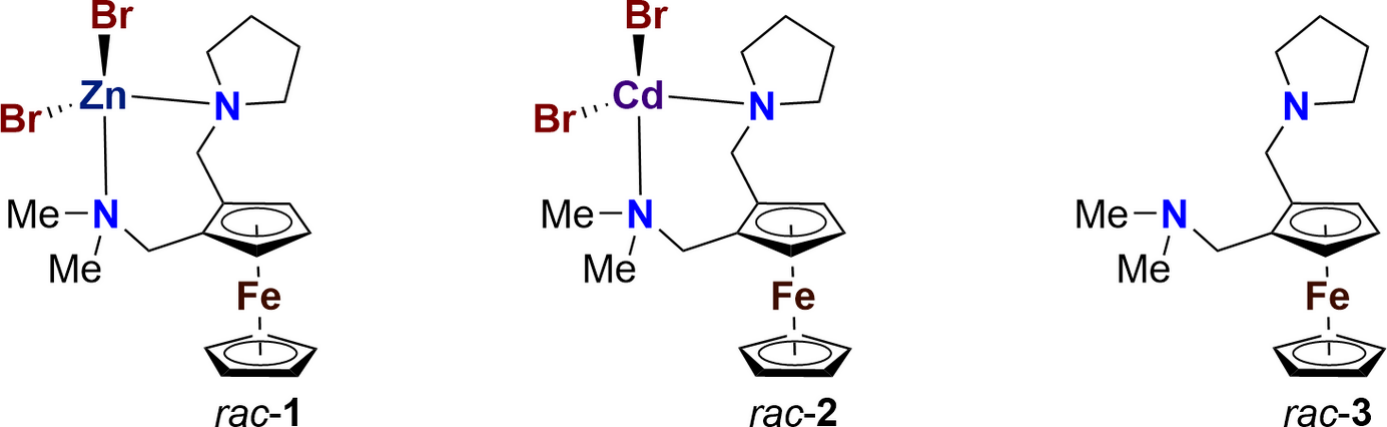


## 2. Structural commentary

**Figure 1:**
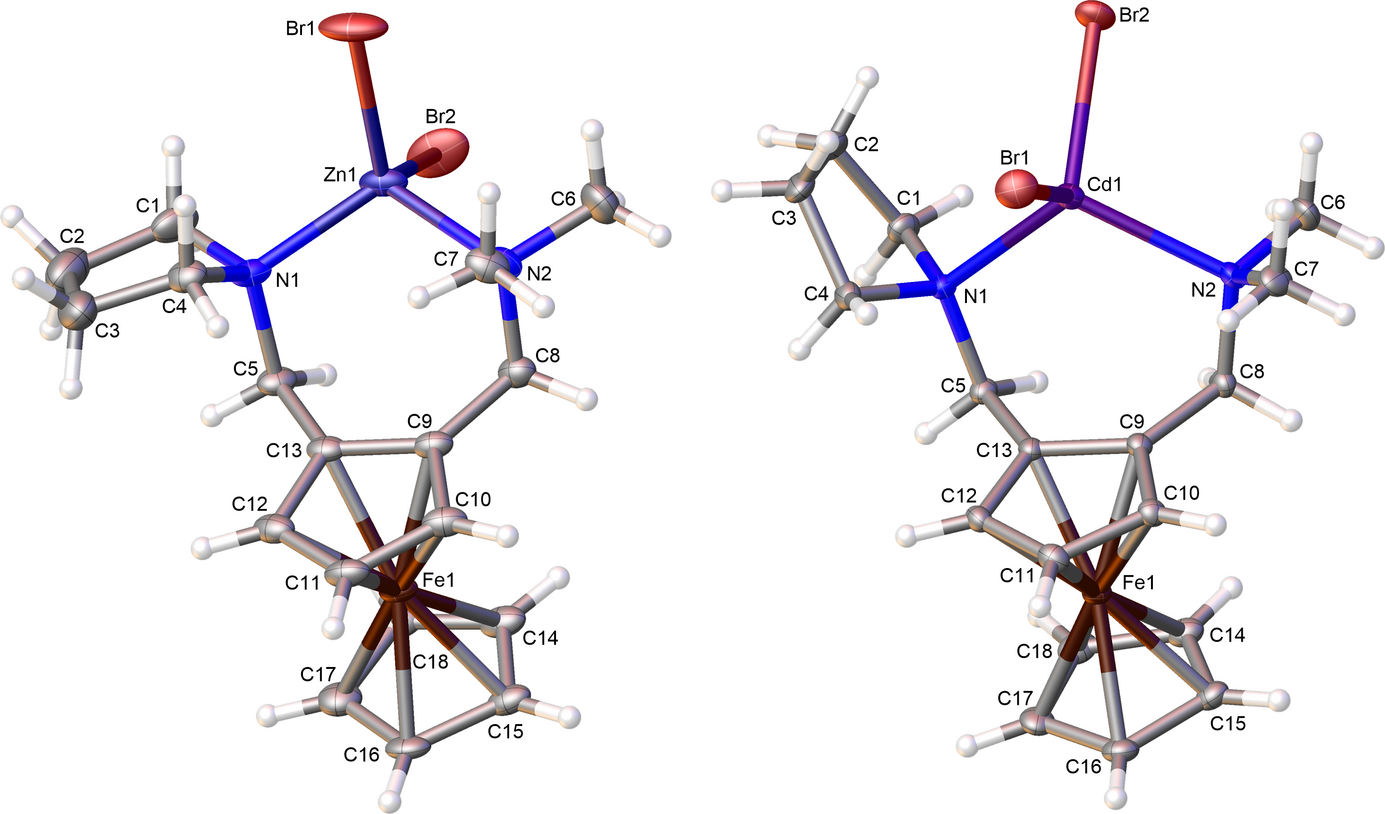
Mol­ecular structures of *rac*-**1** (left) and *rac*-**2** (right), showing the atom labelling and 50% probability displacement ellipsoids.

The zinc(II) complex *rac*-**1** crystallizes at room temperature from acetone in the form of yellow blocks in the monoclinic space group *P*2_1_*/n*. Compound *rac*-**1** exhibits a tetra­hedral coordination geometry around the zinc center with two bromide anions and *rac*-**3** as the bidentate ferrocenyl ligand. The mol­ecular structure of *rac*-**1** is illustrated in Fig. 1 ▸ (left), and selected bond lengths, bond angles as well as torsion angles are given in Table 1 ▸.

The cadmium(II) complex *rac*-**2** crystallizes at room temperature from acetone in the form of yellow blocks in the ortho­rhom­bic space group *P*2_1_2_1_2_1_. Compound *rac*-**2** also exhibits a tetra­hedral coordination geometry around the cadmium center with two bromide anions and *rac*-**3** as the bidentate ferrocenyl ligand. The mol­ecular structure of *rac*-**2** is illustrated in Fig. 1 ▸ as well (right), and selected bond lengths, bond angles as well as torsion angles are given in Table 1 ▸.

The tetra­hedral geometry at the metal center, which is present in both complexes, can be identified by the angles around the zinc center [Br1—Zn1—Br2: 113.88 (4)°, N1—Zn1—N2: 110.45 (8)°, Br1—Zn1—N2: 107.20 (7)°, Br2—Zn1—N1: 106.69 (7)°] and the angles around the cadmium center [Br1—Cd1—Br2: 113.236 (11)°, N1—Cd1—N2: 112.60 (3)°, Br1—Cd1—N2: 107.86 (3)°, Br2—Cd1—N1: 109.79 (2)°], which are close to 109°. It is noticeable that the angles between Br1—*M*—Br2 (*M* = metal center) and N1—*M*—N2 are larger than those between Br1—*M*—N2 and Br2—*M*—N1. The cyclo­penta­dienyl rings are arranged nearly parallel to each other; however, the structure of complex *rac*-**2** exhibits a slightly greater offset between the two rings [H10—C10—C15—H15: 3.2749 (4)° (*rac*-**1**), H10—C10—C15—H15: −7.4408 (9)° (*rac*-**2**)].

The bond lengths between the transition metal center and the coordinating domains of the two complexes differ the most. All of these bonds are shorter in complex *rac*-**1** [N1—Zn1: 2.050 (2) Å, N2—Zn1: 2.077 (2) Å, Br1—Zn1: 2.3560 (10) Å, Br2—Zn1: 2.3606 (10) Å] compared to complex *rac*-**2** [N1—Cd1: 2.3148 (10) Å, N2—Cd1: 2.3250 (11) Å, Br1—Cd1: 2.5544 (5) Å, Br2—Cd1: 2.5685 (5) Å]. This observation is consistent with the increasing size of the transition metal from zinc to cadmium.

The main difference between the two complexes presented is the orientation of the functional groups. For example, the pyrrolidine substituent in complex *rac*-**1** is bent slightly downwards [C3—C4—C13—C9: −171.3 (2)°], whereas in complex *rac*-**2** it is bent upwards [C3—C4—C13—C9: 35.4 (2)°]. The bromido ligands are also orientated differently. In the zinc(II) complex, Br1 is positioned vertically above the metal center [C5—N1—Zn1—Br1: −167.24 (13)°], while Br2 is bent back from the ferrocene unit [C5—N1—Zn1—Br2: 68.03 (14)°]. In the cadmium(II) complex, Br1 is orientated less towards the ferrocene unit [C5—N1—Cd1—Br1: −108.21 (6)°], while Br2 is only slightly bent backwards [C5—N1—Cd1—Br2: 127.62 (6)°]. Furthermore, the methyl groups of the di­methyl­amino­methyl substituent are also orientated differently [N1—Zn1—N2—C6: 166.39 (17)° (*rac*-**1**), N1—Zn1—N2—C7: −76.41 (14)° (*rac*-**1**), N1—Cd1—N2—C6: 108.68 (8)° (*rac*-**2**), N1—Cd1—N2—C7: −132.05 (7)° (*rac*-**2**)]. All these observations are consistent with the different arrangements of the nitro­gen groups around the metal centers. In the zinc(II) complex *rac*-**1**, they are more bent towards the ferrocene unit [C4—N1—C5—C13: −52.5 (2)°, C7—N2—C8—C9: 50.0 (2)°], whereas the nitro­gen substituents in the cadmium(II) complex *rac*-**2** are further away from the ferrocene unit and positioned more laterally [C4—N1—C5—C13: −70.55 (10)°, C7—N2—C8—C9: 70.56 (10)°].

## 3. Supra­molecular features

**Figure 2:**
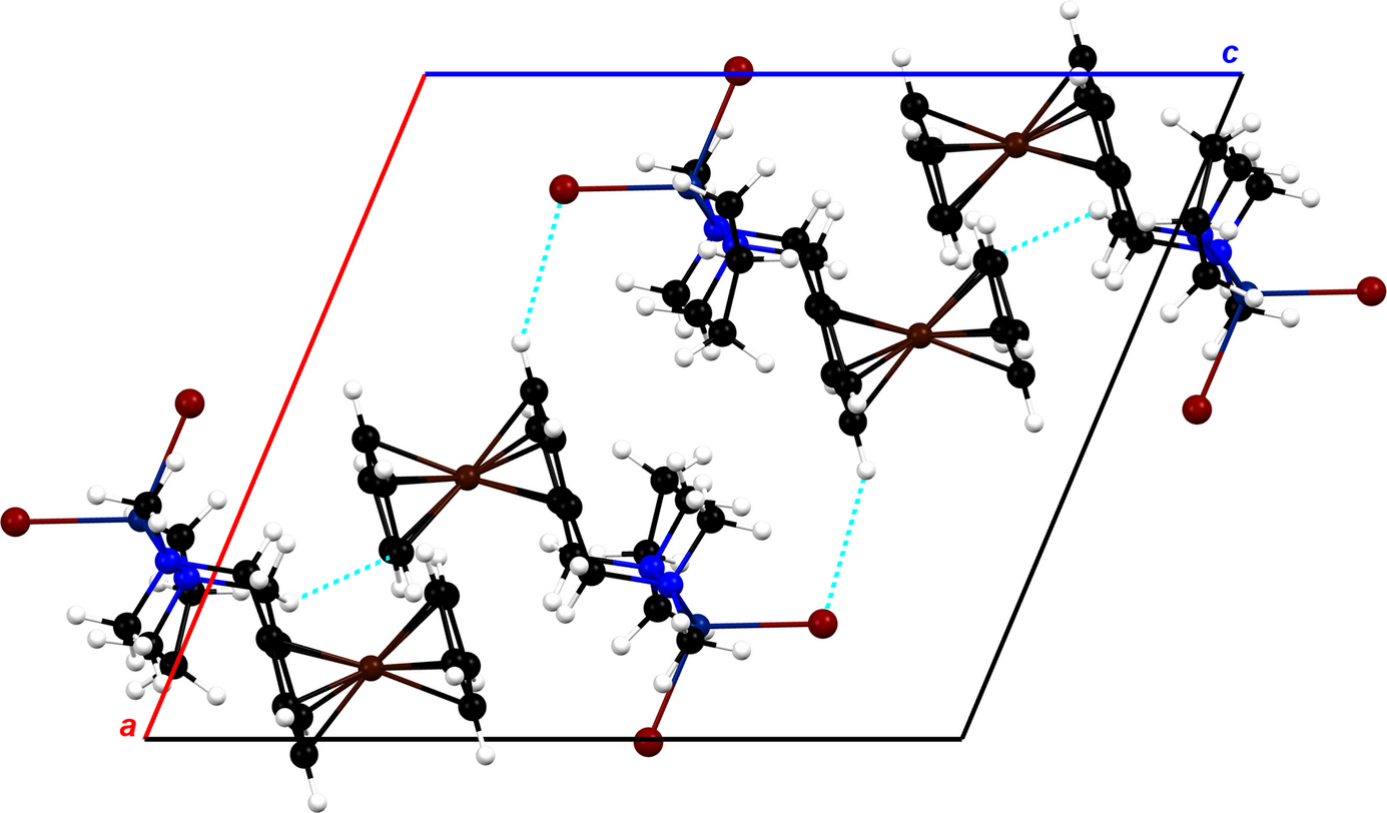
The mol­ecular packing of *rac*-**1** viewed along the *b* axis with the unit cell shown as a black outline. Short contacts are shown as dashed blue lines.

**Figure 3:**
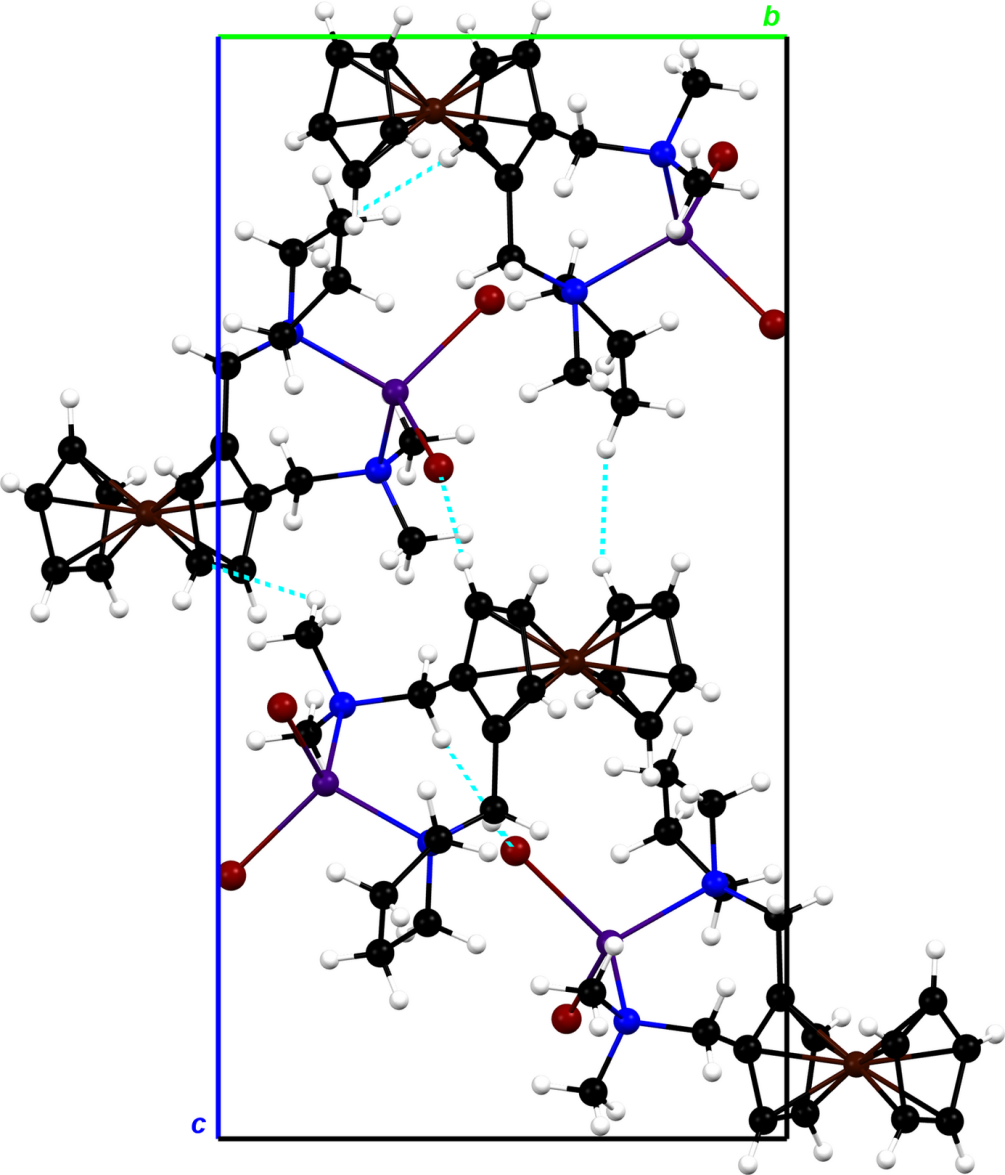
The mol­ecular packing of *rac*-**2** viewed along the *a* axis with the unit cell shown as a black outline. Short contacts are shown as dashed blue lines.

Despite the use of a racemic mixture of the chiral ligand *rac*-**3***, rac*-**1** crystallizes in a centrosymmetric space group, while *rac*-**2** crystallizes in a chiral space group. Therefore, the investigation of the close inter­molecular contacts that determine the arrangement of mol­ecules in the crystal packing, is of particular inter­est. The crystal packing between four mol­ecules of complex *rac*-**1** is shown in Fig. 2 ▸. Short inter­molecular contacts corresponding to hydrogen bonds can be seen, which originate from the bromido ligands or the carbon atoms of the cyclo­penta­dienyl rings. Furthermore, Fig. 3 ▸ shows the crystal packing of complex *rac*-**2**, which also exhibits short inter­molecular contacts. In addition to hydrogen bonds, inter­molecular H⋯H inter­actions can be observed. These inter­actions involve the hydrogen atoms of the pyrrolidine substituent of *rac*-**2**, which could explain the different configurations of the pyrrolidine rings in both complexes. The main difference between the crystal structures is the formation of parallel layers of compound *rac*-**1**, whereas the orientation of the mol­ecules of compound *rac*-**2** seems to be more random.

**Figure 4:**
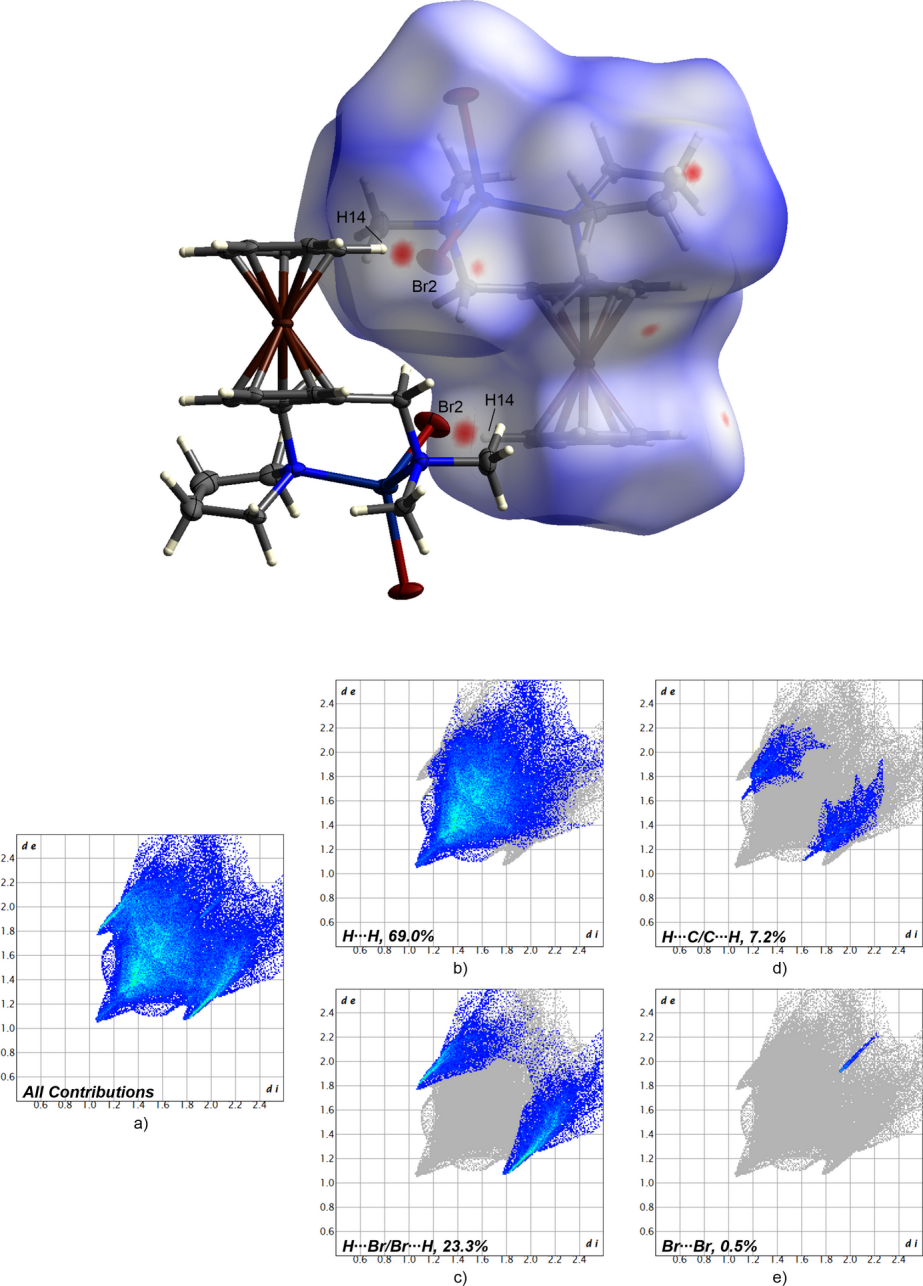
Hirshfeld surface analysis showing close contacts, and two-dimensional fingerprint plots for *rac*-**1**; (*a*) all contributions and (*b*)–(*e*) contributions between specific inter­acting atom pairs (blue areas).

**Figure 5:**
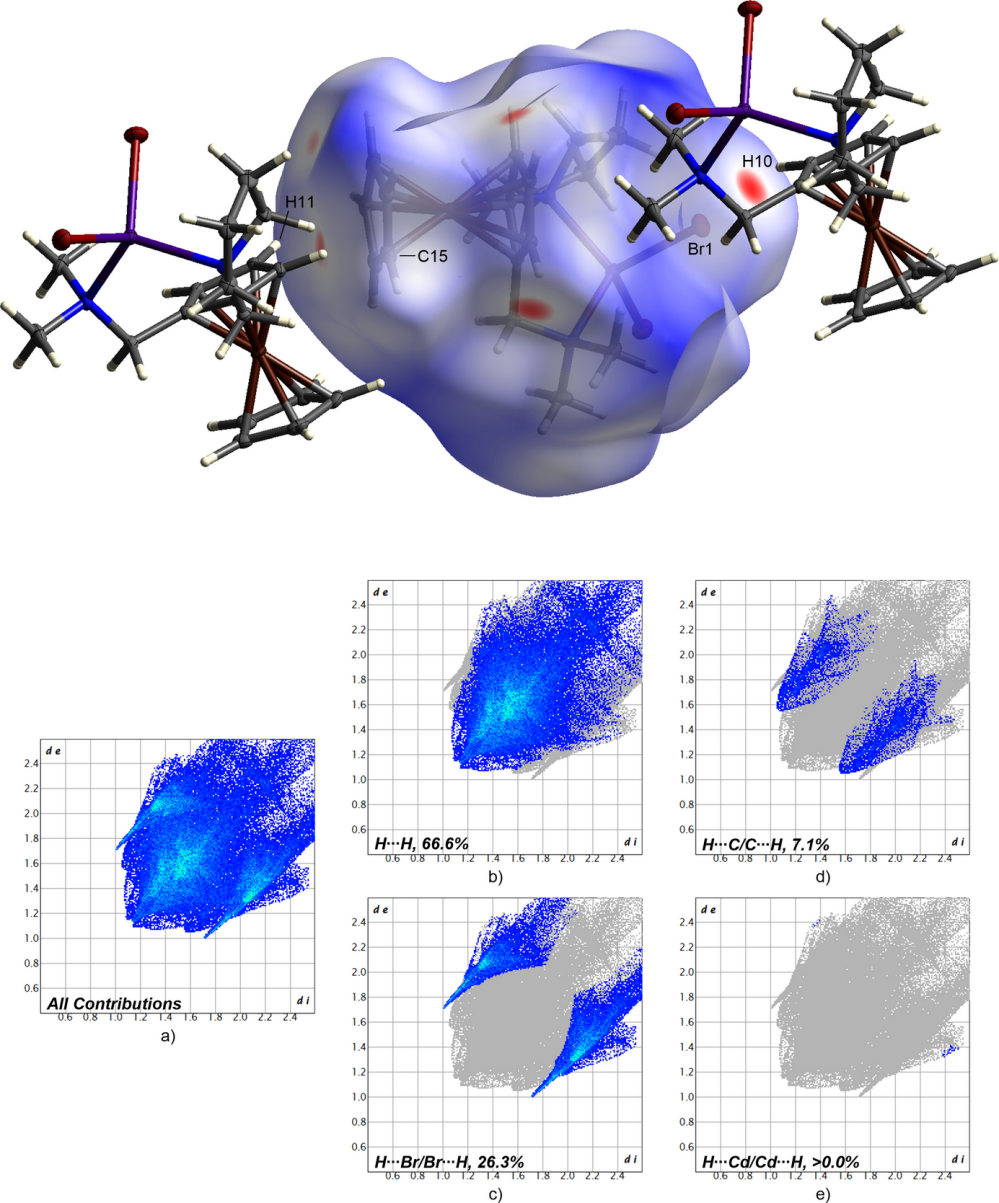
Hirshfeld surface analysis showing close contacts, and two-dimensional fingerprint plots for *rac*-**2**; (*a*) all contributions and (*b*)–(*e*) contributions between specific inter­acting atom pairs (blue areas).

To better understand the inter­molecular inter­actions and to investigate which inter­molecular inter­action is dominating the packing of *rac*-**1** and *rac*-**2**, Hirshfeld surface analyses (Spackman & Jayatilaka, 2009 ▸) were carried out. The surfaces and the corresponding fingerprint plots (McKinnon *et al.*, 2007 ▸) were calculated using *CrystalExplorer21* (Spackman *et al.*, 2021 ▸). Fig. 4 ▸ illustrates the Hirshfeld surface for the zinc(II) complex *rac*-**1** mapped over *d*_norm_ in the range from −0.0606 to 1.6786 arbitrary units. For the cadmium(II) complex *rac*-**2**, the surface shown in Fig. 5 ▸ was mapped over *d*_norm_ in the range from −0.1546 to 1.7781 arbitrary units. The red areas represent the closest contacts. In compound *rac*-**1**, especially the Br⋯H inter­actions are highlighted by red spots. In contrast, for compound *rac*-**2**, the hydrogen bonds, that originate not only from the bromido ligands but also from the carbon atoms of the cyclo­penta­dienyl rings, dominate.

The contributions of the respective inter­molecular inter­actions are visualized by the two-dimensional fingerprint plots shown for complex *rac*-**1** in Fig. 6 and for complex *rac*-**2** in Fig. 7. In both crystal structures, the H⋯H inter­actions can be identified as the most significant inter­actions with 69.0% for the packing of *rac*-**1** and 66.6% for *rac*-**2**. These are followed by the H⋯Br inter­actions, which contribute 23.3% to the packing of complex *rac*-**1** and 26.3% to the packing of complex *rac*-**2**. In addition, C⋯H inter­actions are also relevant for the respective crystal packing. These contribute to the packing to nearly the same extent, with a percentage of 7.2% (*rac*-**1**) and 7.1% (*rac*-**2**), respectively. Furthermore, in the crystal packing of complex *rac*-**1**, Br⋯Br inter­actions contribute to the packing with a small percentage of 0.5%. In contrast, the weakest inter­actions in the packing of complex *rac*-**2** could be identified as those between Cd and H (>0.0%). However, the latter inter­actions contribute less to the crystal packings of complex *rac*-**1** and *rac*-**2**. Based on this analysis, the H⋯H inter­action could be identified as the most significant inter­action of the crystal packing of both compounds.

## 4. Database survey

A search of the Cambridge Structural Database (Groom *et al.*, 2016 ▸; WebCSD February 2026) revealed several structures of similar transition-metal complexes. For example, there are two nickel(II) halide complexes with 1,2-bis­(*N*,*N*-di­methyl­amino­meth­yl)ferrocene as ligand which is very similar to ligand *rac*-**3** used in this work. The nickel center is coordinated by the bidentate ferrocene-based ligand and two chlorides in the first (ZAMNIO; Butler *et al.*, 2026 ▸) and two bromides in the second solid-state structure (MUCRUA; Butler *et al.*, 2026 ▸). Furthermore, there are solid-state structures that are more similar to complexes *rac*-**1** and *rac*-**2** in terms of the transition metal. While the first complex is a zinc(II) bromide complex with a ferrocene terpyridyl ligand (VUDHIN; Wu *et al.*, 2017 ▸), the second complex contains cadmium(II) as the central metal cation with the same bidentate ligand (OGEYEG; Wu *et al.*, 2017 ▸). In the structures with 1,2-bis­(*N*,*N*-di­methyl­amino­meth­yl)ferrocene as ligand, the transition metal adopts a tetra­hedral coordination geometry, like at complexes *rac*-**1** and *rac*-**2** at hand.

## 5. Synthesis and crystallization

For the synthesis of ligand *rac*-**3**, *N*,*N*-di­methyl­amino­methyl­ferrocene (243.13 g mol^−1^, 0.99 mL, *ρ* = 1.23 g mL^−1^, 1.22 g, 5.00 mmol, 1.00 eq.) was added to 15 mL of dried diethyl ether at 273 K under inert conditions. After adding *tert*-butyl­lithium (64.06 g mol^−1^, 3.42 mL, *c* = 1.90 mol L^−1^ in *n*-pentane, 416 mg, 6.50 mmol, 1.30 eq.) at 273 K, the mixture was stirred for 10 min at 273 K and then for 30 min at room temperature. Subsequently, the solution was cooled to 193 K and 1-methyl­idenepyrrolidin-1-ium chloride (119.59 g mol^−1^, 837 mg, 7.00 mmol, 1.40 eq.) was added. The solution was allowed to warm to room temperature over 4 h. It was then diluted with water and the pH was adjusted with KOH to pH = 14. After phase separation, the aqueous phase was extracted with diethyl ether (3×20mL). The organic phases were dried over MgSO_4_ and the solvent was removed under reduced pressure. After purification by column chromatography, ligand *rac*-**3** (326.27 g mol^−1^, 393 mg, 1.20 mmol, 24%.) was isolated as a brown oil.

To crystallize *rac*-**1**, ligand *rac*-**3** (326.27 g mol^−1^, 10.0 mg, 0.03 mmol, 1.00 eq.) was dissolved in acetone (4 mL). Zinc bromide (225.19 g mol^−1^, 6.8 mg, 0.03 mmol, 1.00 eq.) was then added. Subsequently, the solvent was slowly evaporated at room temperature. Product *rac*-**1** crystallized in the form of yellow blocks, which were suitable for X-ray diffraction.

To crystallize *rac*-**2**, ligand *rac*-**3** (326.27 g mol^−1^, 10.0 mg, 0.03 mmol, 1.00 eq.) was dissolved in acetone (4 mL). Cadmium bromide (272.22 g mol^−1^, 8.2 mg, 0.03 mmol, 1.00 eq.) was then added. Subsequently, the solvent was slowly evaporated at room temperature. Product *rac*-**2** crystallized in the form of yellow blocks, which were suitable for X-ray diffraction.

Characterization of the ligand *rac*-**3**:

GC/EI-MS [353 K (1 min) – 573 K (32 min) at 30 K min^−1^] (70 eV, *t*_R_ = 15.624 min) *m*/*z* (%) = 326 (11) (*M*^+^), 281 (100) [(*M* – NMe_2_ – H)^+^], 268 (4) [(*M* – CH_2_NMe_2_)^+^], 255 (86) [(*M* – Pyrr – H)^+^], 213 (52) [(*M* – CH_2_Pyrr – 2Me)^+^], 121 (79) (CpFe^+^), 58 (13) (CH_2_NMe_2_^+^).

^1^H-NMR (600 MHz, C_6_D_6_) δ = 1.57–1.66 (*m*, 4H; Pyrr-NCH_2_C*H*_2_), 2.19 [*s*, 6H; N(C*H*_3_)_2_], 2.43–2.51 (*m*, 2H; Pyrr-NC*H*_2_CH_2_), 2.51–2.59 (*m*, 2H; Pyrr-NC*H*_2_CH_2_), 3.16, 3.40 [AB-system, *J*_AB_ = 12.7, 2H; C*H*_2_N(CH_3_)_2_], 3.32, 3.59 (AB-system, *J*_AB_ = 12.8, 2H; C*H*_2_Pyrr-N), 3.93 (*s*, 5H; C_5_*H*_5_), 3.95–3.97 (*m*, 1H; Cp-C*H*), 4.19 (*s*, 1H; Cp-C*H*), 4.23 (*s*, 1H; Cp-C*H*) ppm.

^13^C{^1^H}-NMR (151 MHz, C_6_D_6_) δ = 24.0 (2C; Pyrr-NCH_2_*C*H_2_), 45.4 [2C; N(*C*H_3_)_2_], 53.8 (1C; C*H*_2_Pyrr-N), 54.4 (2C; Pyrr-N*C*H_2_CH_2_), 57.8 [1C; C*H*_2_N(CH_3_)_2_], 66.8 (1C; Cp-*C*H), 69.5 (5C; *C*_5_H_5_), 70.3 (1C; Cp-*C*H), 70.6 (1C; Cp-*C*H), 84.3 (1C; Cp-*C*_quar_), 85.7 (1C; Cp-*C*_quar_) ppm.

## 6. Refinement

Crystal data, data collection and structure refinement details are summarized in Table 2 ▸. For both compounds, the H atoms were positioned geometrically (C—H = 0.95–0.99 Å) and refined using a riding model, with *U*_iso_(H) = 1.2*U*_eq_(C) for CH_2_ and CH hydrogen atoms and *U*_iso_(H) = 1.5*U*_eq_(C) for CH_3_ hydrogen atoms. For refinement of complex *rac*-**2**, twin law (–1, 0, 0, 0, −1, 0, 0, 0, −1) was applied.

## Supplementary Material

Crystal structure: contains datablock(s) rac-1, rac-2, New_Global_Publ_Block. DOI: 10.1107/S2056989026004688/tx2110sup1.cif

CCDC references: 2551916, 2551915

Additional supporting information:  crystallographic information; 3D view; checkCIF report

## Figures and Tables

**Table 1:** Bond geometry (Å, °; *M* = metal center)

Bond lengths	*rac*-**1**	*rac*-**2**
N1—*M*	2.050 (2)	2.3148 (10)
N2—*M*	2.077 (2)	2.3250 (11)
Br1—*M*	2.3560 (10)	2.5544 (5)
Br2—*M*	2.3606 (10)	2.5685 (5)
		
Bond angles	*rac*-**1**	*rac*-**2**
Br1—*M*—Br2	113.88 (4)	113.236 (11)
N1—*M*—N2	110.45 (8)	112.60 (3)
Br1—*M*—N2	107.20 (7)	107.86 (3)
Br2—*M*—N1	106.69 (7)	109.79 (2)
		
Torsion angles	*rac*-**1**	*rac*-**2**
C3—C4—C13—C9	–171.3 (2)	35.4 (2)
C5—N1—*M*—Br1	–167.24 (13)	–108.21 (6)
C5—N1—*M*—Br2	68.03 (14)	127.62 (6)
N1—*M*—N2—C6	166.39 (17)	108.68 (8)
N1—*M*—N2—C7	–76.41 (14)	–132.05 (7)
C4—N1—C5—C13	–52.5 (2)	–70.55 (10)
C7—N2—C8—C9	50.0 (2)	70.56 (10)
C1—C2—C3—C4	4.1 (2)	–5.11 (10)
H10—C10—C15—H15	3.2749 (4)	–7.4408 (9)

**Table 2:** Experimental details

	rac-**1**	rac-**2**
Crystal data		
Chemical formula	[FeZnBr_2_(C_5_H_5_)(C_13_H_22_N_2_)]	[FeCdBr_2_(C_5_H_5_)(C_13_H_22_N_2_)]
*M*_r_	551.47	598.48
Crystal system, space group	Monoclinic, *P*2_1_/*n*	Orthorhombic, *P*2_1_2_1_2_1_
Temperature (K)	100	100
*a*, *b*, *c* (Å)	13.5397 (11), 10.4169 (9), 15.3223 (14)	9.804 (2), 10.502 (3), 20.326 (5)
α, β, γ (°)	90, 112.846 (4), 90	90, 90, 90
*V* (Å^3^)	1991.6 (3)	2092.9 (8)
*Z*	4	4
Radiation type	Mo *K*α	Mo *K*α
μ (mm^−1^)	5.96	5.54
Crystal size (mm)	0.61 × 0.33 × 0.18	0.17 × 0.15 × 0.15

Data collection		
Diffractometer	Bruker D8 VENTURE area detector	Bruker D8 VENTURE area detector
Absorption correction	Multi-scan (*SADABS*; Krause *et al.*, 2015 ▸)	Multi-scan (*SADABS*; Krause *et al.*, 2015 ▸)
*T*_min_, *T*_max_	0.196, 0.563	0.459, 0.568
No. of measured, independent and observed [*I* > 2σ(*I*)] reflections	144623, 4425, 4276	291242, 11159, 10771
*R*_int_	0.063	0.057
(sin θ/λ)_max_ (Å^−1^)	0.643	0.861

Refinement		
*R*[*F*^2^ > 2σ(*F*^2^)], *wR*(*F*^2^), *S*	0.027, 0.069, 1.04	0.015, 0.032, 1.04
No. of reflections	4425	11159
No. of parameters	294	320
H-atom treatment	H-atom parameters constrained	H-atom parameters constrained
Δρ_max_, Δρ_min_ (e Å^−3^)	1.22, −0.48	0.64, −0.42
Absolute structure	–	Hooft *et al.* (2010 ▸)
Absolute structure parameter	–	−0.0058 (13)

Computer programs: *APEX6* and *SAINT* (Bruker, 2016 ▸), *SHELXT* (Sheldrick, 2015 ▸), *OLEX2.refine* (Bourhis *et al.*, 2015 ▸) and *OLEX2* (Dolomanov *et al.*, 2009 ▸).

## supplementary crystallographic information

### rac-Dibromido[1-(dimethylaminomethyl)-2-(pyrrolidin-1-ylmethyl)\ ferrocene]zinc(II) (rac-1) Crystal data

**Table:**

[FeZnBr_2_(C_5_H_5_)(C_13_H_22_N_2_)]	*F*(000) = 1097.663
*M**_r_* = 551.47	*D*_x_ = 1.839 Mg m^−^^3^
Monoclinic, *P*2_1_/*n*	Mo *K*α radiation, λ = 0.71073 Å
*a* = 13.5397 (11) Å	Cell parameters from 9845 reflections
*b* = 10.4169 (9) Å	θ = 2.4–29.6°
*c* = 15.3223 (14) Å	µ = 5.96 mm^−^^1^
β = 112.846 (4)°	*T* = 100 K
*V* = 1991.6 (3) Å^3^	Block, clear yellow
*Z* = 4	0.61 × 0.33 × 0.18 mm

### rac-Dibromido[1-(dimethylaminomethyl)-2-(pyrrolidin-1-ylmethyl)\ ferrocene]zinc(II) (rac-1) Data collection

**Table:**

Bruker D8 VENTURE area detector diffractometer	4425 independent reflections
Radiation source: microfocus sealed X-ray tube, Incoatec Iµs	4276 reflections with *I* > 2σ(*I*)
HELIOS mirror optics monochromator	*R*_int_ = 0.063
Detector resolution: 10.4167 pixels mm^-1^	θ_max_ = 27.2°, θ_min_ = 2.6°
ω and φ scans	*h* = −17→17
Absorption correction: multi-scan (SADABS; Krause *et al.*, 2015)	*k* = −13→13
*T*_min_ = 0.196, *T*_max_ = 0.563	*l* = −19→19
144623 measured reflections	

### rac-Dibromido[1-(dimethylaminomethyl)-2-(pyrrolidin-1-ylmethyl)\ ferrocene]zinc(II) (rac-1) Refinement

**Table:**

Refinement on *F*^2^	0 restraints
Least-squares matrix: full	42 constraints
*R*[*F*^2^ > 2σ(*F*^2^)] = 0.027	H-atom parameters constrained
*wR*(*F*^2^) = 0.069	*w* = 1/[σ^2^(*F*_o_^2^) + (0.0291*P*)^2^ + 4.1878*P*] where *P* = (*F*_o_^2^ + 2*F*_c_^2^)/3
*S* = 1.04	(Δ/σ)_max_ = −0.0002
4425 reflections	Δρ_max_ = 1.22 e Å^−^^3^
294 parameters	Δρ_min_ = −0.48 e Å^−^^3^

### rac-Dibromido[1-(dimethylaminomethyl)-2-(pyrrolidin-1-ylmethyl)\ ferrocene]zinc(II) (rac-1) Fractional atomic coordinates and isotropic or equivalent isotropic displacement parameters (Å^2^)

**Table:**

	*x*	*y*	*z*	*U*_iso_*/*U*_eq_	
Zn1	0.83060 (6)	0.55352 (8)	0.61828 (5)	0.0234 (3)	
Br1	0.82659 (6)	0.56794 (11)	0.77031 (5)	0.0438 (4)	
Br2	1.00453 (6)	0.56586 (9)	0.61791 (6)	0.0435 (3)	
Fe1	0.60674 (3)	0.48115 (3)	0.25968 (2)	0.01804 (10)	
N2	0.76792 (15)	0.3755 (2)	0.56325 (14)	0.0208 (4)	
N1	0.74283 (16)	0.6978 (2)	0.53186 (13)	0.0196 (4)	
C9	0.65123 (18)	0.4361 (2)	0.39863 (16)	0.0185 (5)	
C13	0.64094 (18)	0.5724 (2)	0.38531 (16)	0.0185 (5)	
C7	0.6694 (2)	0.3467 (3)	0.57945 (18)	0.0249 (5)	
H7a	0.6865 (4)	0.342 (2)	0.64767 (18)	0.0374 (8)*	
H7b	0.6164 (6)	0.4145 (11)	0.5512 (13)	0.0374 (8)*	
H7c	0.6401 (9)	0.2641 (10)	0.5502 (13)	0.0374 (8)*	
C5	0.72546 (19)	0.6706 (2)	0.43034 (16)	0.0203 (5)	
H5a	0.70508 (19)	0.7513 (2)	0.39354 (16)	0.0244 (6)*	
H5b	0.79367 (19)	0.6405 (2)	0.42786 (16)	0.0244 (6)*	
C14	0.7193 (2)	0.4056 (3)	0.21604 (18)	0.0254 (5)	
H14	0.7801 (2)	0.3565 (3)	0.25353 (18)	0.0305 (6)*	
C8	0.74846 (19)	0.3637 (3)	0.46043 (16)	0.0214 (5)	
H8a	0.81171 (19)	0.3966 (3)	0.45018 (16)	0.0257 (6)*	
H8b	0.73959 (19)	0.2719 (3)	0.44222 (16)	0.0257 (6)*	
C15	0.61545 (19)	0.3561 (3)	0.15997 (17)	0.0237 (5)	
H15	0.59481 (19)	0.2684 (3)	0.15340 (17)	0.0285 (6)*	
C12	0.53302 (19)	0.5987 (3)	0.32237 (16)	0.0229 (5)	
H12	0.50374 (19)	0.6813 (3)	0.30116 (16)	0.0275 (6)*	
C17	0.6101 (2)	0.5768 (3)	0.14389 (18)	0.0273 (5)	
H17	0.5854 (2)	0.6616 (3)	0.12479 (18)	0.0327 (7)*	
C16	0.5486 (2)	0.4624 (3)	0.11578 (16)	0.0238 (5)	
H16	0.4752 (2)	0.4576 (3)	0.07444 (16)	0.0285 (6)*	
C11	0.47712 (19)	0.4803 (3)	0.29699 (17)	0.0233 (5)	
H11	0.40414 (19)	0.4699 (3)	0.25576 (17)	0.0280 (6)*	
C4	0.6404 (2)	0.7302 (3)	0.54268 (17)	0.0237 (5)	
H4a	0.5833 (2)	0.6685 (3)	0.50716 (17)	0.0284 (6)*	
H4b	0.6506 (2)	0.7280 (3)	0.61022 (17)	0.0284 (6)*	
C10	0.54923 (19)	0.3805 (3)	0.34390 (16)	0.0224 (5)	
H10	0.53264 (19)	0.2915 (3)	0.33973 (16)	0.0269 (6)*	
C18	0.7154 (2)	0.5411 (3)	0.20580 (18)	0.0276 (6)	
H18	0.7735 (2)	0.5984 (3)	0.23537 (18)	0.0331 (7)*	
C1	0.8031 (2)	0.8221 (3)	0.56020 (19)	0.0313 (6)	
H1a	0.8370 (2)	0.8293 (3)	0.63004 (19)	0.0376 (7)*	
H1b	0.8597 (2)	0.8273 (3)	0.53449 (19)	0.0376 (7)*	
C6	0.8510 (2)	0.2803 (3)	0.6167 (2)	0.0337 (6)	
H6a	0.9164 (7)	0.2960 (14)	0.6054 (13)	0.0505 (9)*	
H6b	0.8668 (14)	0.2885 (15)	0.6846 (3)	0.0505 (9)*	
H6c	0.8247 (8)	0.1935 (3)	0.5956 (12)	0.0505 (9)*	
C2	0.7211 (3)	0.9280 (3)	0.5192 (2)	0.0430 (8)	
H2a	0.7355 (3)	1.0007 (3)	0.5640 (2)	0.0516 (9)*	
H2b	0.7225 (3)	0.9598 (3)	0.4588 (2)	0.0516 (9)*	
C3	0.6111 (3)	0.8649 (3)	0.5027 (2)	0.0388 (7)	
H3a	0.5648 (3)	0.8621 (3)	0.4344 (2)	0.0466 (8)*	
H3b	0.5735 (3)	0.9126 (3)	0.5366 (2)	0.0466 (8)*	

### rac-Dibromido[1-(dimethylaminomethyl)-2-(pyrrolidin-1-ylmethyl)\ ferrocene]zinc(II) (rac-1) Atomic displacement parameters (Å^2^)

**Table:**

	*U*^11^	*U*^22^	*U*^33^	*U*^12^	*U*^13^	*U*^23^
Zn1	0.0120 (5)	0.0434 (7)	0.0148 (5)	−0.0036 (4)	0.0050 (4)	−0.0041 (4)
Br1	0.0268 (5)	0.0904 (11)	0.0142 (4)	−0.0048 (5)	0.0079 (4)	−0.0062 (5)
Br2	0.0135 (4)	0.0710 (8)	0.0490 (6)	−0.0114 (4)	0.0152 (4)	−0.0205 (5)
Fe1	0.01387 (16)	0.0287 (2)	0.01229 (16)	−0.00098 (13)	0.00587 (12)	−0.00400 (13)
N2	0.0151 (9)	0.0270 (11)	0.0188 (9)	0.0024 (8)	0.0049 (8)	0.0032 (8)
N1	0.0183 (9)	0.0274 (11)	0.0140 (9)	−0.0064 (8)	0.0074 (7)	−0.0046 (8)
C9	0.0136 (10)	0.0290 (12)	0.0138 (10)	−0.0025 (9)	0.0064 (8)	−0.0034 (9)
C13	0.0158 (10)	0.0298 (13)	0.0114 (10)	−0.0004 (9)	0.0068 (8)	−0.0020 (9)
C7	0.0212 (12)	0.0330 (14)	0.0220 (12)	−0.0034 (10)	0.0099 (9)	0.0057 (10)
C5	0.0232 (11)	0.0264 (12)	0.0136 (10)	−0.0041 (9)	0.0097 (9)	−0.0014 (9)
C14	0.0175 (11)	0.0417 (15)	0.0188 (11)	0.0000 (10)	0.0092 (9)	−0.0075 (10)
C8	0.0176 (11)	0.0281 (12)	0.0187 (11)	0.0008 (9)	0.0072 (9)	−0.0029 (9)
C15	0.0190 (11)	0.0358 (14)	0.0182 (11)	−0.0023 (10)	0.0092 (9)	−0.0095 (10)
C12	0.0182 (11)	0.0364 (14)	0.0150 (10)	0.0049 (10)	0.0073 (9)	−0.0021 (10)
C17	0.0312 (14)	0.0358 (15)	0.0184 (12)	−0.0006 (11)	0.0136 (10)	−0.0013 (10)
C16	0.0219 (12)	0.0390 (14)	0.0104 (10)	−0.0012 (10)	0.0061 (9)	−0.0044 (10)
C11	0.0144 (10)	0.0398 (15)	0.0153 (11)	−0.0013 (10)	0.0051 (9)	−0.0054 (10)
C4	0.0220 (11)	0.0320 (13)	0.0195 (11)	0.0016 (10)	0.0106 (9)	−0.0045 (10)
C10	0.0169 (11)	0.0356 (14)	0.0164 (10)	−0.0058 (10)	0.0085 (9)	−0.0038 (10)
C18	0.0238 (12)	0.0428 (15)	0.0217 (12)	−0.0074 (11)	0.0148 (10)	−0.0075 (11)
C1	0.0377 (15)	0.0310 (14)	0.0259 (13)	−0.0153 (12)	0.0129 (11)	−0.0090 (11)
C6	0.0284 (13)	0.0337 (15)	0.0322 (14)	0.0105 (12)	0.0044 (11)	0.0059 (12)
C2	0.069 (2)	0.0286 (15)	0.0341 (16)	−0.0047 (15)	0.0229 (16)	−0.0047 (12)
C3	0.0482 (18)	0.0350 (16)	0.0287 (14)	0.0115 (14)	0.0101 (13)	−0.0040 (12)

### rac-Dibromido[1-(dimethylaminomethyl)-2-(pyrrolidin-1-ylmethyl)\ ferrocene]zinc(II) (rac-1) Geometric parameters (Å, º)

**Table:**

Zn1—Br1	2.3560 (10)	C14—C15	1.429 (3)
Zn1—Br2	2.3606 (10)	C14—C18	1.418 (4)
Zn1—N2	2.077 (2)	C8—H8a	0.9900
Zn1—N1	2.050 (2)	C8—H8b	0.9900
Fe1—C9	2.030 (2)	C15—H15	0.9500
Fe1—C13	2.033 (2)	C15—C16	1.423 (4)
Fe1—C14	2.044 (2)	C12—H12	0.9500
Fe1—C15	2.045 (2)	C12—C11	1.420 (4)
Fe1—C12	2.040 (2)	C17—H17	0.9500
Fe1—C17	2.051 (3)	C17—C16	1.422 (4)
Fe1—C16	2.042 (2)	C17—C18	1.422 (4)
Fe1—C11	2.046 (2)	C16—H16	0.9500
Fe1—C10	2.038 (2)	C11—H11	0.9500
Fe1—C18	2.045 (3)	C11—C10	1.417 (4)
N2—C7	1.479 (3)	C4—H4a	0.9900
N2—C8	1.498 (3)	C4—H4b	0.9900
N2—C6	1.485 (3)	C4—C3	1.521 (4)
N1—C5	1.507 (3)	C10—H10	0.9500
N1—C4	1.498 (3)	C18—H18	0.9500
N1—C1	1.502 (3)	C1—H1a	0.9900
C9—C13	1.434 (3)	C1—H1b	0.9900
C9—C8	1.494 (3)	C1—C2	1.517 (5)
C9—C10	1.431 (3)	C6—H6a	0.9800
C13—C5	1.489 (3)	C6—H6b	0.9800
C13—C12	1.430 (3)	C6—H6c	0.9800
C7—H7a	0.9800	C2—H2a	0.9900
C7—H7b	0.9800	C2—H2b	0.9900
C7—H7c	0.9800	C2—C3	1.556 (5)
C5—H5a	0.9900	C3—H3a	0.9900
C5—H5b	0.9900	C3—H3b	0.9900
C14—H14	0.9500		
			
Br2—Zn1—Br1	113.88 (4)	H5b—C5—H5a	107.8
N2—Zn1—Br1	107.20 (7)	H14—C14—Fe1	126.20 (8)
N2—Zn1—Br2	107.36 (7)	C15—C14—Fe1	69.60 (14)
N1—Zn1—Br1	111.21 (7)	C15—C14—H14	126.05 (17)
N1—Zn1—Br2	106.69 (7)	C18—C14—Fe1	69.72 (15)
N1—Zn1—N2	110.45 (8)	C18—C14—H14	126.05 (15)
C13—Fe1—C9	41.34 (10)	C18—C14—C15	107.9 (2)
C14—Fe1—C9	107.39 (10)	C9—C8—N2	112.15 (19)
C14—Fe1—C13	124.41 (10)	H8a—C8—N2	109.18 (12)
C15—Fe1—C9	123.82 (10)	H8a—C8—C9	109.18 (13)
C15—Fe1—C13	161.29 (10)	H8b—C8—N2	109.18 (13)
C15—Fe1—C14	40.91 (9)	H8b—C8—C9	109.18 (13)
C12—Fe1—C9	69.22 (10)	H8b—C8—H8a	107.9
C12—Fe1—C13	41.12 (9)	C14—C15—Fe1	69.50 (14)
C12—Fe1—C14	161.43 (11)	H15—C15—Fe1	126.31 (8)
C12—Fe1—C15	156.11 (10)	H15—C15—C14	126.25 (17)
C17—Fe1—C9	157.30 (10)	C16—C15—Fe1	69.51 (14)
C17—Fe1—C13	121.23 (11)	C16—C15—C14	107.5 (2)
C17—Fe1—C14	68.53 (11)	C16—C15—H15	126.25 (14)
C17—Fe1—C15	68.66 (11)	C13—C12—Fe1	69.15 (13)
C17—Fe1—C12	107.15 (11)	H12—C12—Fe1	126.72 (7)
C16—Fe1—C9	160.59 (11)	H12—C12—C13	125.83 (15)
C16—Fe1—C13	156.60 (11)	C11—C12—Fe1	69.87 (14)
C16—Fe1—C14	68.51 (10)	C11—C12—C13	108.3 (2)
C16—Fe1—C15	40.75 (11)	C11—C12—H12	125.83 (14)
C16—Fe1—C12	120.78 (10)	H17—C17—Fe1	126.50 (8)
C16—Fe1—C17	40.66 (11)	C16—C17—Fe1	69.35 (14)
C11—Fe1—C9	69.11 (9)	C16—C17—H17	126.26 (16)
C11—Fe1—C13	69.03 (9)	C18—C17—Fe1	69.45 (15)
C11—Fe1—C14	156.77 (11)	C18—C17—H17	126.26 (17)
C11—Fe1—C15	120.69 (10)	C18—C17—C16	107.5 (2)
C11—Fe1—C12	40.67 (11)	C15—C16—Fe1	69.74 (13)
C11—Fe1—C17	123.61 (11)	C17—C16—Fe1	69.99 (14)
C11—Fe1—C16	106.76 (10)	C17—C16—C15	108.6 (2)
C10—Fe1—C9	41.19 (9)	H16—C16—Fe1	126.13 (7)
C10—Fe1—C13	69.12 (10)	H16—C16—C15	125.72 (14)
C10—Fe1—C14	121.72 (11)	H16—C16—C17	125.72 (16)
C10—Fe1—C15	107.00 (11)	C12—C11—Fe1	69.45 (14)
C10—Fe1—C12	68.51 (11)	H11—C11—Fe1	126.75 (7)
C10—Fe1—C17	160.12 (10)	H11—C11—C12	125.98 (14)
C10—Fe1—C16	123.54 (10)	C10—C11—Fe1	69.38 (13)
C10—Fe1—C11	40.60 (10)	C10—C11—C12	108.0 (2)
C18—Fe1—C9	121.81 (10)	C10—C11—H11	125.98 (14)
C18—Fe1—C13	107.73 (10)	H4a—C4—N1	110.59 (12)
C18—Fe1—C14	40.60 (12)	H4b—C4—N1	110.59 (12)
C18—Fe1—C15	68.51 (11)	H4b—C4—H4a	108.7
C18—Fe1—C12	124.55 (11)	C3—C4—N1	105.7 (2)
C18—Fe1—C17	40.62 (11)	C3—C4—H4a	110.59 (15)
C18—Fe1—C16	68.25 (10)	C3—C4—H4b	110.59 (14)
C18—Fe1—C11	160.74 (12)	C9—C10—Fe1	69.11 (13)
C18—Fe1—C10	157.65 (11)	C11—C10—Fe1	70.02 (14)
C7—N2—Zn1	111.47 (16)	C11—C10—C9	108.6 (2)
C8—N2—Zn1	112.20 (15)	H10—C10—Fe1	126.73 (7)
C8—N2—C7	110.97 (18)	H10—C10—C9	125.72 (15)
C6—N2—Zn1	105.65 (16)	H10—C10—C11	125.72 (14)
C6—N2—C7	108.1 (2)	C14—C18—Fe1	69.68 (15)
C6—N2—C8	108.2 (2)	C17—C18—Fe1	69.93 (15)
C5—N1—Zn1	110.35 (15)	C17—C18—C14	108.6 (2)
C4—N1—Zn1	115.04 (15)	H18—C18—Fe1	126.25 (7)
C4—N1—C5	112.12 (18)	H18—C18—C14	125.72 (15)
C1—N1—Zn1	109.37 (16)	H18—C18—C17	125.72 (17)
C1—N1—C5	108.08 (19)	H1a—C1—N1	110.49 (13)
C1—N1—C4	101.3 (2)	H1b—C1—N1	110.49 (13)
C13—C9—Fe1	69.44 (13)	H1b—C1—H1a	108.7
C8—C9—Fe1	127.69 (16)	C2—C1—N1	106.2 (2)
C8—C9—C13	127.1 (2)	C2—C1—H1a	110.49 (16)
C10—C9—Fe1	69.70 (13)	C2—C1—H1b	110.49 (17)
C10—C9—C13	107.4 (2)	H6a—C6—N2	109.5
C10—C9—C8	125.4 (2)	H6b—C6—N2	109.5
C9—C13—Fe1	69.22 (13)	H6b—C6—H6a	109.5
C5—C13—Fe1	127.69 (16)	H6c—C6—N2	109.5
C5—C13—C9	126.8 (2)	H6c—C6—H6a	109.5
C12—C13—Fe1	69.74 (13)	H6c—C6—H6b	109.5
C12—C13—C9	107.6 (2)	H2a—C2—C1	110.72 (16)
C12—C13—C5	125.5 (2)	H2b—C2—C1	110.72 (17)
H7a—C7—N2	109.5	H2b—C2—H2a	108.8
H7b—C7—N2	109.5	C3—C2—C1	105.1 (2)
H7b—C7—H7a	109.5	C3—C2—H2a	110.72 (16)
H7c—C7—N2	109.5	C3—C2—H2b	110.72 (17)
H7c—C7—H7a	109.5	C2—C3—C4	103.9 (2)
H7c—C7—H7b	109.5	H3a—C3—C4	110.97 (15)
C13—C5—N1	113.04 (18)	H3a—C3—C2	110.97 (17)
H5a—C5—N1	108.98 (13)	H3b—C3—C4	110.97 (15)
H5a—C5—C13	108.98 (13)	H3b—C3—C2	110.97 (16)
H5b—C5—N1	108.98 (12)	H3b—C3—H3a	109.0
H5b—C5—C13	108.98 (13)		
			
Zn1—N2—C8—C9	−75.46 (16)	N2—C8—C9—C13	74.9 (2)
Zn1—N1—C5—C13	77.16 (16)	N2—C8—C9—C10	−102.8 (2)
Zn1—N1—C4—C3	159.53 (18)	N1—C5—C13—C9	−77.2 (2)
Zn1—N1—C1—C2	−160.76 (19)	N1—C5—C13—C12	100.9 (2)
Fe1—C9—C13—C5	−122.20 (13)	N1—C4—C3—C2	−28.5 (2)
Fe1—C9—C13—C12	59.38 (14)	N1—C1—C2—C3	21.6 (2)
Fe1—C9—C8—N2	166.6 (2)	C9—C13—C12—C11	0.0 (2)
Fe1—C9—C10—C11	−59.07 (15)	C9—C8—N2—C7	50.0 (2)
Fe1—C13—C9—C8	122.34 (13)	C9—C8—N2—C6	168.4 (2)
Fe1—C13—C9—C10	−59.60 (14)	C9—C10—C11—C12	−0.4 (2)
Fe1—C13—C5—N1	−168.3 (2)	C13—C9—C10—C11	0.4 (2)
Fe1—C13—C12—C11	59.06 (15)	C13—C5—N1—C4	−52.5 (2)
Fe1—C14—C15—C16	59.36 (15)	C13—C5—N1—C1	−163.3 (2)
Fe1—C14—C18—C17	−59.31 (16)	C13—C12—C11—C10	0.2 (2)
Fe1—C15—C14—C18	−59.43 (16)	C5—N1—C4—C3	−73.3 (2)
Fe1—C15—C16—C17	59.42 (15)	C5—N1—C1—C2	79.1 (2)
Fe1—C12—C13—C9	−59.06 (14)	C5—C13—C9—C8	0.1 (3)
Fe1—C12—C13—C5	122.49 (13)	C5—C13—C9—C10	178.2 (2)
Fe1—C12—C11—C10	58.83 (15)	C5—C13—C12—C11	−178.4 (2)
Fe1—C17—C16—C15	−59.26 (15)	C14—C15—C16—C17	0.1 (2)
Fe1—C17—C18—C14	59.15 (15)	C14—C18—C17—C16	0.0 (2)
Fe1—C16—C15—C14	−59.35 (15)	C8—C9—C13—C12	−178.3 (2)
Fe1—C16—C17—C18	59.21 (16)	C8—C9—C10—C11	178.5 (2)
Fe1—C11—C12—C13	−58.61 (14)	C15—C14—C18—C17	0.0 (2)
Fe1—C11—C10—C9	58.51 (14)	C15—C16—C17—C18	−0.1 (2)
Fe1—C10—C9—C13	59.44 (14)	C12—C13—C9—C10	−0.2 (2)
Fe1—C10—C9—C8	−122.47 (13)	C16—C15—C14—C18	−0.1 (2)
Fe1—C10—C11—C12	−58.88 (15)	C4—N1—C1—C2	−38.9 (2)
Fe1—C18—C14—C15	59.35 (15)	C4—C3—C2—C1	4.1 (2)
Fe1—C18—C17—C16	−59.15 (15)	C1—N1—C4—C3	41.7 (2)

### rac-Dibromido[1-(dimethylaminomethyl)-2-(pyrrolidin-1-ylmethyl)\ ferrocene]cadmium(II) (rac-2) Crystal data

**Table:**

[FeCdBr_2_(C_5_H_5_)(C_13_H_22_N_2_)]	*D*_x_ = 1.899 Mg m^−^^3^
*M**_r_* = 598.48	Mo *K*α radiation, λ = 0.71073 Å
Orthorhombic, *P*2_1_2_1_2_1_	Cell parameters from 9365 reflections
*a* = 9.804 (2) Å	θ = 2.2–37.8°
*b* = 10.502 (3) Å	µ = 5.54 mm^−^^1^
*c* = 20.326 (5) Å	*T* = 100 K
*V* = 2092.9 (8) Å^3^	Block, clear yellow
*Z* = 4	0.17 × 0.15 × 0.15 mm
*F*(000) = 1165.116	

### rac-Dibromido[1-(dimethylaminomethyl)-2-(pyrrolidin-1-ylmethyl)\ ferrocene]cadmium(II) (rac-2) Data collection

**Table:**

Bruker D8 VENTURE area detector diffractometer	11159 independent reflections
Radiation source: microfocus sealed X-ray tube, Incoatec Iµs	10771 reflections with *I* > 2σ(*I*)
HELIOS mirror optics monochromator	*R*_int_ = 0.057
Detector resolution: 10.4167 pixels mm^-1^	θ_max_ = 37.7°, θ_min_ = 2.0°
ω and φ scans	*h* = −16→16
Absorption correction: multi-scan (SADABS; Krause *et al.*, 2015)	*k* = −18→18
*T*_min_ = 0.459, *T*_max_ = 0.568	*l* = −34→34
291242 measured reflections	

### rac-Dibromido[1-(dimethylaminomethyl)-2-(pyrrolidin-1-ylmethyl)\ ferrocene]cadmium(II) (rac-2) Refinement

**Table:**

Refinement on *F*^2^	42 constraints
Least-squares matrix: full	H-atom parameters constrained
*R*[*F*^2^ > 2σ(*F*^2^)] = 0.015	*w* = 1/[σ^2^(*F*_o_^2^) + (0.0089*P*)^2^ + 0.7418*P*] where *P* = (*F*_o_^2^ + 2*F*_c_^2^)/3
*wR*(*F*^2^) = 0.032	(Δ/σ)_max_ = 0.0001
*S* = 1.04	Δρ_max_ = 0.64 e Å^−^^3^
11159 reflections	Δρ_min_ = −0.41 e Å^−^^3^
320 parameters	Absolute structure: Hooft *et al.* (2010)
0 restraints	Absolute structure parameter: −0.0058 (13)

### rac-Dibromido[1-(dimethylaminomethyl)-2-(pyrrolidin-1-ylmethyl)\ ferrocene]cadmium(II) (rac-2) Fractional atomic coordinates and isotropic or equivalent isotropic displacement parameters (Å^2^)

**Table:**

	*x*	*y*	*z*	*U*_iso_*/*U*_eq_	
Cd1	0.254301 (19)	0.188601 (16)	0.677351 (9)	0.01446 (5)	
Br2	0.33680 (3)	0.02257 (3)	0.761163 (14)	0.02191 (7)	
Br1	0.05181 (3)	0.11252 (3)	0.608742 (15)	0.02481 (8)	
Fe1	0.32868 (4)	0.62236 (3)	0.567501 (18)	0.01373 (8)	
N1	0.19181 (9)	0.37363 (9)	0.73139 (4)	0.01347 (15)	
C12	0.14120 (11)	0.55061 (10)	0.59185 (6)	0.01462 (17)	
H12	0.06653 (11)	0.59599 (10)	0.61021 (6)	0.0175 (2)*	
N2	0.43788 (10)	0.21868 (9)	0.60664 (5)	0.01630 (16)	
C17	0.32688 (13)	0.81605 (11)	0.58190 (6)	0.0209 (2)	
H17	0.25098 (13)	0.86632 (11)	0.59493 (6)	0.0250 (2)*	
C13	0.24755 (12)	0.48882 (9)	0.62818 (5)	0.01270 (14)	
C9	0.33922 (11)	0.43087 (9)	0.58130 (5)	0.01270 (16)	
C10	0.28778 (11)	0.45821 (10)	0.51653 (5)	0.01441 (17)	
H10	0.32768 (11)	0.43149 (10)	0.47624 (5)	0.0173 (2)*	
C5	0.26299 (11)	0.48505 (10)	0.70149 (5)	0.01409 (16)	
H5a	0.22548 (11)	0.56447 (10)	0.72049 (5)	0.01691 (19)*	
H5b	0.36117 (11)	0.48108 (10)	0.71269 (5)	0.01691 (19)*	
C11	0.16671 (11)	0.53225 (10)	0.52315 (5)	0.01581 (17)	
H11	0.11239 (11)	0.56391 (10)	0.48808 (5)	0.0190 (2)*	
C14	0.52060 (12)	0.69060 (12)	0.58564 (6)	0.01874 (19)	
H14	0.59606 (12)	0.64294 (12)	0.60155 (6)	0.0225 (2)*	
C16	0.36489 (13)	0.78615 (11)	0.51582 (6)	0.0194 (2)	
H16	0.31870 (13)	0.81318 (11)	0.47718 (6)	0.0233 (2)*	
C15	0.48462 (12)	0.70841 (11)	0.51798 (6)	0.0185 (2)	
H15	0.53185 (12)	0.67462 (11)	0.48110 (6)	0.0222 (2)*	
C2	0.10371 (14)	0.28455 (13)	0.83139 (7)	0.0246 (2)	
H2a	0.13527 (14)	0.19579 (13)	0.83746 (7)	0.0295 (3)*	
H2b	0.07126 (14)	0.31781 (13)	0.87419 (7)	0.0295 (3)*	
C8	0.46610 (10)	0.35796 (10)	0.59686 (5)	0.01469 (17)	
H8a	0.50789 (10)	0.39309 (10)	0.63731 (5)	0.0176 (2)*	
H8b	0.53222 (10)	0.36858 (10)	0.56046 (5)	0.0176 (2)*	
C18	0.42328 (14)	0.75695 (12)	0.62495 (6)	0.0208 (2)	
H18	0.42263 (14)	0.76115 (12)	0.67163 (6)	0.0250 (3)*	
C4	0.03991 (11)	0.38733 (11)	0.72830 (6)	0.01767 (19)	
H4a	0.01146 (11)	0.47487 (11)	0.74005 (6)	0.0212 (2)*	
H4b	0.00520 (11)	0.36676 (11)	0.68381 (6)	0.0212 (2)*	
C1	0.21845 (12)	0.36794 (12)	0.80395 (6)	0.0189 (2)	
H1a	0.30869 (12)	0.32948 (12)	0.81302 (6)	0.0227 (2)*	
H1b	0.21557 (12)	0.45416 (12)	0.82359 (6)	0.0227 (2)*	
C7	0.40243 (14)	0.15941 (11)	0.54247 (6)	0.0217 (2)	
H7a	0.3866 (12)	0.0681 (2)	0.54872 (12)	0.0326 (3)*	
H7b	0.3196 (7)	0.1992 (8)	0.5250 (3)	0.0326 (3)*	
H7c	0.4777 (5)	0.1718 (10)	0.51141 (19)	0.0326 (3)*	
C3	−0.01109 (13)	0.29116 (12)	0.77897 (7)	0.0234 (2)	
H3a	−0.02575 (13)	0.20680 (12)	0.75842 (7)	0.0281 (3)*	
H3b	−0.09780 (13)	0.32010 (12)	0.79896 (7)	0.0281 (3)*	
C6	0.56305 (14)	0.15719 (14)	0.63277 (7)	0.0258 (3)	
H6a	0.5873 (7)	0.1960 (8)	0.6750 (3)	0.0387 (4)*	
H6b	0.5463 (4)	0.0660 (3)	0.6390 (6)	0.0387 (4)*	
H6c	0.6381 (4)	0.1690 (10)	0.6015 (3)	0.0387 (4)*	

### rac-Dibromido[1-(dimethylaminomethyl)-2-(pyrrolidin-1-ylmethyl)\ ferrocene]cadmium(II) (rac-2) Atomic displacement parameters (Å^2^)

**Table:**

	*U*^11^	*U*^22^	*U*^33^	*U*^12^	*U*^13^	*U*^23^
Cd1	0.01563 (9)	0.01182 (8)	0.01593 (9)	0.00027 (8)	−0.00165 (8)	0.00051 (7)
Br2	0.02612 (16)	0.01943 (14)	0.02019 (14)	0.00332 (12)	−0.00653 (12)	0.00448 (12)
Br1	0.02324 (15)	0.02825 (17)	0.02294 (15)	−0.00560 (13)	−0.00902 (12)	−0.00041 (13)
Fe1	0.01543 (18)	0.01014 (17)	0.01563 (18)	−0.00174 (15)	0.00029 (15)	0.00102 (14)
N1	0.0142 (4)	0.0126 (3)	0.0137 (4)	0.0002 (3)	0.0019 (3)	0.0017 (3)
C12	0.0126 (4)	0.0116 (4)	0.0197 (5)	0.0009 (3)	0.0010 (3)	0.0007 (3)
N2	0.0166 (4)	0.0119 (4)	0.0204 (4)	0.0045 (3)	0.0022 (3)	0.0014 (3)
C17	0.0247 (5)	0.0105 (4)	0.0275 (5)	−0.0027 (4)	0.0031 (4)	−0.0010 (4)
C13	0.0133 (3)	0.0095 (3)	0.0153 (4)	−0.0001 (4)	0.0021 (4)	0.0007 (3)
C9	0.0135 (4)	0.0095 (4)	0.0150 (4)	0.0000 (3)	0.0012 (3)	−0.0002 (3)
C10	0.0158 (4)	0.0123 (4)	0.0151 (4)	−0.0002 (3)	−0.0001 (3)	−0.0015 (3)
C5	0.0152 (4)	0.0118 (4)	0.0153 (4)	−0.0017 (3)	0.0016 (3)	−0.0001 (3)
C11	0.0149 (4)	0.0137 (4)	0.0188 (4)	−0.0001 (4)	−0.0033 (4)	−0.0004 (3)
C14	0.0177 (4)	0.0181 (4)	0.0204 (5)	−0.0057 (4)	−0.0027 (3)	0.0033 (4)
C16	0.0213 (5)	0.0142 (4)	0.0227 (5)	−0.0029 (4)	−0.0008 (4)	0.0062 (4)
C15	0.0173 (4)	0.0194 (5)	0.0189 (5)	−0.0045 (4)	0.0017 (4)	0.0042 (4)
C2	0.0302 (6)	0.0214 (5)	0.0222 (6)	0.0017 (4)	0.0097 (5)	0.0062 (4)
C8	0.0121 (4)	0.0138 (4)	0.0182 (4)	0.0012 (3)	0.0012 (3)	0.0001 (3)
C18	0.0267 (6)	0.0175 (5)	0.0183 (5)	−0.0086 (4)	−0.0008 (4)	−0.0024 (4)
C4	0.0135 (4)	0.0152 (4)	0.0243 (5)	0.0004 (3)	0.0045 (4)	0.0017 (4)
C1	0.0240 (5)	0.0195 (5)	0.0132 (4)	0.0003 (4)	0.0023 (3)	0.0012 (4)
C7	0.0297 (6)	0.0136 (4)	0.0219 (5)	0.0006 (4)	0.0053 (4)	−0.0037 (4)
C3	0.0211 (5)	0.0200 (5)	0.0292 (6)	−0.0028 (4)	0.0106 (4)	0.0047 (4)
C6	0.0209 (5)	0.0250 (6)	0.0316 (6)	0.0106 (4)	0.0040 (5)	0.0084 (5)

### rac-Dibromido[1-(dimethylaminomethyl)-2-(pyrrolidin-1-ylmethyl)\ ferrocene]cadmium(II) (rac-2) Geometric parameters (Å, º)

**Table:**

Cd1—Br2	2.5685 (5)	C10—C11	1.4254 (16)
Cd1—Br1	2.5544 (5)	C5—H5a	0.9900
Cd1—N1	2.3148 (10)	C5—H5b	0.9900
Cd1—N2	2.3250 (11)	C11—H11	0.9500
Fe1—C12	2.0473 (12)	C14—H14	0.9500
Fe1—C17	2.0553 (13)	C14—C15	1.4320 (17)
Fe1—C13	2.0299 (10)	C14—C18	1.4264 (19)
Fe1—C9	2.0332 (11)	C16—H16	0.9500
Fe1—C10	2.0508 (12)	C16—C15	1.4305 (17)
Fe1—C11	2.0566 (12)	C15—H15	0.9500
Fe1—C14	2.0471 (12)	C2—H2a	0.9900
Fe1—C16	2.0466 (12)	C2—H2b	0.9900
Fe1—C15	2.0415 (12)	C2—C1	1.5309 (18)
Fe1—C18	2.0547 (12)	C2—C3	1.551 (2)
N1—C5	1.4919 (14)	C8—H8a	0.9900
N1—C4	1.4975 (14)	C8—H8b	0.9900
N1—C1	1.4989 (15)	C18—H18	0.9500
C12—H12	0.9500	C4—H4a	0.9900
C12—C13	1.4330 (15)	C4—H4b	0.9900
C12—C11	1.4316 (16)	C4—C3	1.5268 (16)
N2—C8	1.5020 (15)	C1—H1a	0.9900
N2—C7	1.4863 (16)	C1—H1b	0.9900
N2—C6	1.4849 (16)	C7—H7a	0.9800
C17—H17	0.9500	C7—H7b	0.9800
C17—C16	1.4289 (18)	C7—H7c	0.9800
C17—C18	1.4298 (19)	C3—H3a	0.9900
C13—C9	1.4444 (15)	C3—H3b	0.9900
C13—C5	1.4983 (15)	C6—H6a	0.9800
C9—C10	1.4388 (15)	C6—H6b	0.9800
C9—C8	1.4945 (15)	C6—H6c	0.9800
C10—H10	0.9500		
			
Br1—Cd1—Br2	113.236 (11)	C8—C9—C10	125.98 (9)
N1—Cd1—Br2	109.79 (2)	C9—C10—Fe1	68.71 (6)
N1—Cd1—Br1	108.42 (2)	H10—C10—Fe1	127.11 (3)
N2—Cd1—Br2	104.98 (3)	H10—C10—C9	125.84 (6)
N2—Cd1—Br1	107.86 (3)	C11—C10—Fe1	69.91 (6)
N2—Cd1—N1	112.60 (3)	C11—C10—C9	108.32 (9)
C17—Fe1—C12	108.79 (5)	C11—C10—H10	125.84 (6)
C13—Fe1—C12	41.15 (4)	C13—C5—N1	112.25 (8)
C13—Fe1—C17	126.44 (5)	H5a—C5—N1	109.15 (5)
C9—Fe1—C12	69.40 (4)	H5a—C5—C13	109.15 (5)
C9—Fe1—C17	163.70 (5)	H5b—C5—N1	109.15 (5)
C9—Fe1—C13	41.65 (4)	H5b—C5—C13	109.15 (6)
C10—Fe1—C12	68.73 (4)	H5b—C5—H5a	107.9
C10—Fe1—C17	154.45 (5)	C12—C11—Fe1	69.23 (6)
C10—Fe1—C13	69.49 (4)	C10—C11—Fe1	69.48 (6)
C10—Fe1—C9	41.25 (4)	C10—C11—C12	108.12 (9)
C11—Fe1—C12	40.83 (5)	H11—C11—Fe1	126.92 (3)
C11—Fe1—C17	120.74 (5)	H11—C11—C12	125.94 (6)
C11—Fe1—C13	69.26 (5)	H11—C11—C10	125.94 (6)
C11—Fe1—C9	69.18 (4)	H14—C14—Fe1	126.35 (4)
C11—Fe1—C10	40.61 (4)	C15—C14—Fe1	69.29 (6)
C14—Fe1—C12	155.59 (5)	C15—C14—H14	125.99 (7)
C14—Fe1—C17	68.64 (5)	C18—C14—Fe1	69.94 (7)
C14—Fe1—C13	119.51 (5)	C18—C14—H14	125.99 (7)
C14—Fe1—C9	105.95 (5)	C18—C14—C15	108.02 (11)
C14—Fe1—C10	124.40 (5)	C17—C16—Fe1	69.94 (7)
C14—Fe1—C11	161.77 (5)	H16—C16—Fe1	126.36 (4)
C16—Fe1—C12	126.10 (5)	H16—C16—C17	125.95 (7)
C16—Fe1—C17	40.77 (5)	C15—C16—Fe1	69.32 (6)
C16—Fe1—C13	163.85 (5)	C15—C16—C17	108.09 (11)
C16—Fe1—C9	153.30 (5)	C15—C16—H16	125.95 (7)
C16—Fe1—C10	118.76 (5)	C14—C15—Fe1	69.71 (6)
C16—Fe1—C11	107.20 (5)	C16—C15—Fe1	69.71 (7)
C16—Fe1—C14	68.82 (5)	C16—C15—C14	107.83 (11)
C15—Fe1—C12	162.66 (5)	H15—C15—Fe1	126.07 (4)
C15—Fe1—C17	68.80 (5)	H15—C15—C14	126.08 (7)
C15—Fe1—C13	154.01 (5)	H15—C15—C16	126.08 (7)
C15—Fe1—C9	117.90 (5)	H2b—C2—H2a	108.8
C15—Fe1—C10	105.63 (5)	C1—C2—H2a	110.77 (7)
C15—Fe1—C11	124.46 (5)	C1—C2—H2b	110.77 (7)
C15—Fe1—C14	41.00 (5)	C3—C2—H2a	110.77 (7)
C15—Fe1—C16	40.97 (5)	C3—C2—H2b	110.77 (7)
C18—Fe1—C12	121.41 (5)	C3—C2—C1	104.91 (10)
C18—Fe1—C17	40.71 (5)	C9—C8—N2	111.94 (9)
C18—Fe1—C13	107.87 (5)	H8a—C8—N2	109.22 (6)
C18—Fe1—C9	125.39 (5)	H8a—C8—C9	109.22 (6)
C18—Fe1—C10	162.48 (5)	H8b—C8—N2	109.22 (6)
C18—Fe1—C11	156.09 (5)	H8b—C8—C9	109.22 (6)
C18—Fe1—C14	40.70 (5)	H8b—C8—H8a	107.9
C18—Fe1—C16	68.60 (5)	C17—C18—Fe1	69.66 (7)
C18—Fe1—C15	68.75 (5)	C14—C18—Fe1	69.36 (7)
C5—N1—Cd1	109.95 (6)	C14—C18—C17	108.15 (11)
C4—N1—Cd1	108.90 (7)	H18—C18—Fe1	126.62 (4)
C4—N1—C5	111.89 (8)	H18—C18—C17	125.92 (7)
C1—N1—Cd1	112.79 (7)	H18—C18—C14	125.92 (7)
C1—N1—C5	110.52 (9)	H4a—C4—N1	111.06 (6)
C1—N1—C4	102.62 (8)	H4b—C4—N1	111.06 (6)
H12—C12—Fe1	127.03 (3)	H4b—C4—H4a	109.0
C13—C12—Fe1	68.77 (6)	C3—C4—N1	103.52 (9)
C13—C12—H12	125.83 (6)	C3—C4—H4a	111.06 (7)
C11—C12—Fe1	69.94 (6)	C3—C4—H4b	111.06 (7)
C11—C12—H12	125.83 (6)	C2—C1—N1	104.67 (10)
C11—C12—C13	108.33 (9)	H1a—C1—N1	110.82 (6)
C8—N2—Cd1	110.90 (6)	H1a—C1—C2	110.82 (7)
C7—N2—Cd1	107.73 (7)	H1b—C1—N1	110.82 (6)
C7—N2—C8	109.56 (9)	H1b—C1—C2	110.82 (7)
C6—N2—Cd1	111.07 (8)	H1b—C1—H1a	108.9
C6—N2—C8	108.58 (9)	H7a—C7—N2	109.5
C6—N2—C7	108.97 (10)	H7b—C7—N2	109.5
H17—C17—Fe1	126.61 (4)	H7b—C7—H7a	109.5
C16—C17—Fe1	69.29 (7)	H7c—C7—N2	109.5
C16—C17—H17	126.05 (7)	H7c—C7—H7a	109.5
C18—C17—Fe1	69.62 (7)	H7c—C7—H7b	109.5
C18—C17—H17	126.05 (7)	C4—C3—C2	104.79 (10)
C18—C17—C16	107.90 (11)	H3a—C3—C2	110.79 (7)
C12—C13—Fe1	70.07 (6)	H3a—C3—C4	110.79 (7)
C9—C13—Fe1	69.30 (6)	H3b—C3—C2	110.79 (7)
C9—C13—C12	107.68 (9)	H3b—C3—C4	110.79 (6)
C5—C13—Fe1	125.66 (7)	H3b—C3—H3a	108.9
C5—C13—C12	126.73 (10)	H6a—C6—N2	109.5
C5—C13—C9	125.60 (10)	H6b—C6—N2	109.5
C13—C9—Fe1	69.06 (5)	H6b—C6—H6a	109.5
C10—C9—Fe1	70.03 (6)	H6c—C6—N2	109.5
C10—C9—C13	107.54 (9)	H6c—C6—H6a	109.5
C8—C9—Fe1	125.33 (8)	H6c—C6—H6b	109.5
C8—C9—C13	126.46 (9)		
			
Cd1—N1—C5—C13	50.60 (7)	N1—C5—C13—C12	89.87 (10)
Cd1—N1—C4—C3	75.89 (7)	N1—C5—C13—C9	−90.68 (10)
Cd1—N1—C1—C2	−76.32 (8)	N1—C4—C3—C2	29.97 (10)
Cd1—N2—C8—C9	−48.23 (7)	N1—C1—C2—C3	−21.62 (10)
Fe1—C12—C13—C9	−59.32 (6)	C12—C13—C9—C10	0.11 (9)
Fe1—C12—C13—C5	120.21 (6)	C12—C13—C9—C8	178.96 (8)
Fe1—C12—C11—C10	58.76 (7)	C12—C11—C10—C9	−0.50 (10)
Fe1—C17—C16—C15	−59.02 (7)	N2—C8—C9—C13	88.96 (10)
Fe1—C17—C18—C14	58.86 (7)	N2—C8—C9—C10	−92.40 (10)
Fe1—C13—C12—C11	58.91 (7)	C17—C16—C15—C14	−0.11 (11)
Fe1—C13—C9—C10	−59.70 (7)	C17—C18—C14—C15	−0.03 (11)
Fe1—C13—C9—C8	119.15 (6)	C13—C12—C11—C10	0.57 (10)
Fe1—C13—C5—N1	−179.45 (9)	C13—C9—C10—C11	0.24 (9)
Fe1—C9—C13—C12	59.81 (7)	C13—C5—N1—C4	−70.55 (10)
Fe1—C9—C13—C5	−119.73 (6)	C13—C5—N1—C1	175.76 (9)
Fe1—C9—C10—C11	−58.85 (7)	C9—C13—C12—C11	−0.42 (9)
Fe1—C9—C8—N2	177.75 (9)	C9—C8—N2—C7	70.56 (10)
Fe1—C10—C9—C13	59.09 (6)	C9—C8—N2—C6	−170.53 (10)
Fe1—C10—C9—C8	−119.77 (6)	C10—C9—C13—C5	−179.43 (8)
Fe1—C10—C11—C12	−58.61 (7)	C5—N1—C4—C3	−162.35 (10)
Fe1—C11—C12—C13	−58.19 (7)	C5—N1—C1—C2	160.14 (9)
Fe1—C11—C10—C9	58.11 (7)	C5—C13—C12—C11	179.12 (11)
Fe1—C14—C15—C16	59.51 (7)	C5—C13—C9—C8	−0.58 (12)
Fe1—C14—C18—C17	−59.05 (8)	C11—C10—C9—C8	−178.62 (8)
Fe1—C16—C17—C18	59.11 (8)	C14—C18—C17—C16	−0.04 (11)
Fe1—C16—C15—C14	−59.51 (8)	C16—C15—C14—C18	0.08 (10)
Fe1—C15—C14—C18	−59.43 (7)	C15—C16—C17—C18	0.09 (11)
Fe1—C15—C16—C17	59.40 (8)	C2—C1—N1—C4	40.69 (10)
Fe1—C18—C17—C16	−58.90 (8)	C4—C3—C2—C1	−5.11 (10)
Fe1—C18—C14—C15	59.02 (7)	C1—N1—C4—C3	−43.86 (9)
